# A-GAME: improving the assembly of pooled functional metagenomics sequence data

**DOI:** 10.1186/s12864-017-4369-z

**Published:** 2018-01-12

**Authors:** Matteo Chiara, Antonio Placido, Ernesto Picardi, Luigi Ruggiero Ceci, David Stephen Horner, Graziano Pesole

**Affiliations:** 1 Department of Biosciences, University of Milan, via Celoria 26, 20133 Milan, Italy; 2 Institute of Biomembranes, Bioenergetics and Molecular Biotechnology, Consiglio Nazionale delle Ricerche, via Amendola 165A, 70126 Bari, Italy; 3 Department of Biosciences, Biotechnology and Biopharmaceutics, University of Bari “A. Moro”, via Orabona, 4, 70126 Bari, Italy

**Keywords:** Functional metagenomics, Assembly, Functional annotation, Workflow, Candidate genes, Galaxy

## Abstract

**Background:**

Expression screening of environmental DNA (eDNA) libraries is a popular approach for the identification and characterization of novel microbial enzymes with promising biotechnological properties. In such “functional metagenomics” experiments, inserts, selected on the basis of activity assays, are sequenced with high throughput sequencing technologies. Assembly is followed by gene prediction, annotation and identification of candidate genes that are subsequently evaluated for biotechnological applications.

**Results:**

Here we present A-GAME (A GAlaxy suite for functional MEtagenomics), a web service incorporating state of the art tools and workflows for the analysis of eDNA sequence data. We illustrate the potential of A-GAME workflows using real functional metagenomics data, showing that they outperform alternative metagenomics assemblers. Dedicated tools available in A-GAME allow efficient analysis of pooled libraries and rapid identification of candidate genes, reducing sequencing costs and saving the need for laborious manual annotation.

**Conclusion:**

In conclusion, we believe A-GAME will constitute a valuable resource for the functional metagenomics community.

A-GAME is publicly available at http://beaconlab.it/agame

**Electronic supplementary material:**

The online version of this article (10.1186/s12864-017-4369-z) contains supplementary material, which is available to authorized users.

## Background

Natural ecosystems are home to an almost limitless range of bacteria that have evolved to thrive in often hostile environments. The metabolism and biochemistry of these organisms, underpinned by their genomic sequences, represent a potentially invaluable source of novel biocatalysts and antibiotics with useful physical characteristics.

To partially mitigate difficulties in isolating and obtaining clonal cultures of novel bacteria from extreme environments, many published studies have employed heterologous expression of genes encoded in environmental DNA (eDNA) expression library inserts to identify (and subsequently sequence) genetic loci encoding activities of interest. Novel cellulases, lipases, esterases, proteases, laccases, oxidoreductases, biosurfactants and antibiotics have been sought through such “functional metagenomics” approaches [1, 2]. Much effort has been dedicated to improving heterologous expression screens for eDNA libraries. Indeed, strategies for the selection and modification of heterologous strains [3–5], the choice of optimal cloning vectors for heterologous expression of single gene activities [6], and the random insertion of lab strain-compatible transcriptional and translational signals in eDNA [7, 8] have all been proposed and extensively reviewed elsewhere [9–14].

Automation of expression and screening steps, together with the advent of Next Generation Sequencing (NGS) technologies has vastly increased the potential throughput of functional metagenomics projects, wherein assembled insert sequences are subjected to gene prediction and annotation to identify candidate loci underlying the activities selected through heterologous expression screening.

Assembly of functional metagenomics sequence data should not, in principle, present a particular challenge. Coverage depth is typically high and levels of sequence redundancy within relatively short (typically <45kbp) inserts are expected, in general, to be low. Indeed, costs associated with sequencing can be further reduced by sequencing libraries of pooled inserts [15–17]. Lam et al. used pooled and individually sequenced inserts to provide a detailed evaluation of this approach [16], demonstrating that many inserts could be completely or almost completely assembled in one or two fragments and assigned to their clones of origin through Sanger sequencing of the original insert ends (end-tag sequencing). However, in some cases, more fragmented assemblies emerged as a result of low sequence coverage or other systematic biases.

While assembly strategies optimized for single genomes or whole genome shotgun (WGS) metagenomics projects might be expected to perform comparably with functional genomics sequence data, the requisites of downstream analyses steps differ greatly between single genome, shotgun metagenomics, and functional metagenomics studies.

Here we present A-GAME (A GAlaxy suite for functional MEtagenomics) a powerful and flexible web service implemented within Galaxy [18, 19], a general bioinformatics workflow management system that allows the incorporation of most widely used bioinformatics tools and can be used - even by those lacking programming experience - to build and customize bioinformatics workflows. A-GAME incorporates pre-designed workflows that utilize standard tools for data pre-processing, sequence assembly and annotation; as well as custom utilities dedicated to the analysis of functional metagenomics data. The latter include FosBin, a tool to cluster contigs representing incomplete inserts into groups deriving from single clones, as well as instruments for the synthesis of annotation results - to assist in candidate gene identification and prioritization.

We show, using a real pooled insert functional metagenomics dataset, that preconfigured workflows offered in A-GAME outperform metagenomics assembly pipelines such as MOCAT2 [20] and parallel-META2 [21] in terms of overall quality and completeness of assembly and annotation. Furthermore, we illustrate the use of custom utilities in A-GAME for the identification and prioritization of genes of interest. We suggest that A-GAME will constitute a valuable resource for the functional metagenomics community.

A-GAME is publicly available at http://beaconlab.it/agame

## Implementation

Typical genome assembly and annotation pipelines can be divided into pre-processing, contig assembly, post-processing and annotation phases. A-GAME, which is based on Galaxy release 16.07, follows this convention and provides access to some of the most popular publicly available tools for these tasks as well as featuring a series of ad-hoc custom utilities and scripts for data integration and visualization.

Various combinations of quality trimming and read-merging tools can be employed and sequence data can be screened against Univec, genomes of host strains employed, phiX174, and other databases to eliminate reads deriving from expression vectors, adapters, sequencing reaction spike-ins and other possible sources of contamination prior to assembly. MEGAHIT and MetaVelvet (which were developed for metagenomic data) as well as SPAdes are among the short-read assemblers available through A-GAME. A selection of scaffolding tools can be employed to improve initial assemblies.

Single pass Sanger “end-tag” sequencing [16, 17] can help validate the quality of assemblies and facilitate the association of 2 contigs representing the termini of a single insert to each other and to the insert of origin. A-GAME provides a custom, BLAST-based, utility that automates this operation and assigns end-tag-containing contigs to appropriate clusters.

A further tool, FosBin (see below), employs the K-means clustering algorithm to partition the assembled contigs into the expected number of clusters (number of pooled inserts) based on simple compositional (tetranucleotide frequencies) and coverage (average coverage calculated on windows of 500 bp) metrics - facilitating re-assignment of contigs representing incomplete inserts to their clone of origin. Fosbin can be used in conjunction with the Sanger end-tag sequencing assignment method.

Gene models can be predicted with Glimmer, MetaGeneMark, Prodigal (as incorporated into Prokka) or Augustus. Functional annotations can be generated by PFAMScan. A custom tool integrates annotation information into feature rich files containing inferred protein sequences, concise functional descriptions, and hyperlinks to PFAM entries for detected domains. This output can be queried and filtered by users to retrieve sequences of interest. Selected proteins can be subjected to more comprehensive/thorough functional annotation using the InterPro suite, and compared with the nr database using remote BLASTP or with a local database of over 2500 refseq bacterial proteomes.

A complete list of tools and databases incorporated in A-GAME is shown in Table 1. Further details of custom tools which are made publicly available through Galaxy main tool shed, their deployment in Galaxy and comparisons with existing tools are provided in Additional file 1. A detailed guide to the use of standard and custom facilities in A-GAME is incorporated in a user manual available from the A-GAME homepage.

**Table 1:** List of bioinformatics tools and resources currently incorporated within A-GAME

Quality Trimming	
Tool	Reference
FastQC	[30]
Pear	[31]
Flash	[32]
Trimmomatic	[33]
FastX	[34]
Assembly	
Megahit	[35]
SPAdes	[36]
Abyss	[37]
Velvet	[38]
Meta-Velvet	[39]
Meta-SPAdes	[40]
Gene prediction	
Glimmer	[41]
Augustus	[42]
Prokka (Prodigal)	[43]
Metagenemark	[28]
Functional annotation	
Interpro	[44]
PFAM	[45]
Blast + suite	[46]
Short read mapping	
Bowtie2	[47]
bwa	[48]
Scaffolding	
SSpace	[23]
Sopra	[49]

## Results

### Evaluation of standard workflows using pooled eDNA insert sequencing data

**Fig. 1:**
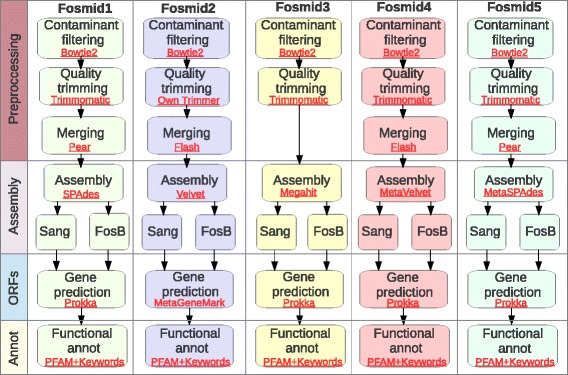
Schematic of workflows for the analysis of metagenomic eDNA data available in A-GAME. Bioinformatics analysis of functional metagenomics data requires pre-processing of raw data, sequence assembly and post-processing of contigs, gene model prediction and functional annotation. A-GAME offers 4 pre-configured workflows, Fosmid1 to Fosmid4, that automate all the steps of the analysis and differ only through the combination of tools used. Schematic of the 4 workflows are represented in the form of flow diagrams, tools used to perform individual steps are reported in red

It is widely accepted that “optimal” data pre-processing and assembly strategies differ between individual sequencing datasets. Accordingly, a series of 5 workflows encompassing pre-processing (quality trimming and read pair merging), assembly, gene model prediction and functional annotation were created using the Galaxy workflow editor in A-GAME (Fig. 1).

**Fig. 2:**
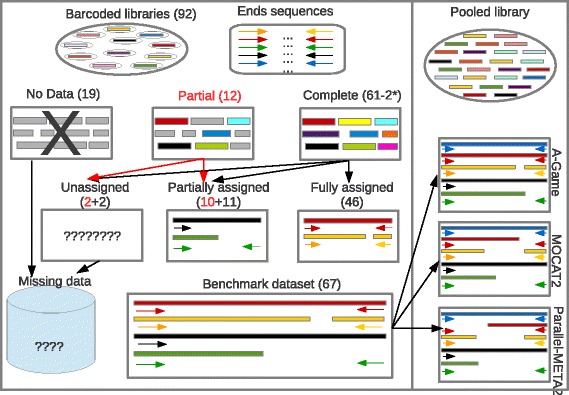
Re-assembly of barcoded sequence data from Lam et al... dataset. Assemblies of bar-coded sequence data from Lam et al. were used to evaluate results achieved by different tool and pipelines in the analysis of pooled sequencing data. Of the 92 clones subjected to individual sequencing by Lam et al. [16] showed lack of coverage or absence of end tags sequences and were therefore discarded from these analyses. Of the 61 inserts for which sequencing information for both “end-tags” was available (“complete”, Indicated in black), 46 were matched correctly to both ends (Fully assigned), 11 were matched with only 1 out of 2 ends (Partially assigned) and 2 did not show any similarity to available insert termini sequences. Of the twelve clones for which only “partial” (single end-tag, indicated in red) sequencing information is available ten were recognized and assigned with the proper end tag while 2 are missing. Only assigned and partially assigned contigs were included in the benchmark dataset used for the evaluation

To evaluate the performance of these pipelines, we utilized published data from pooled and individual high throughput sequencing of 92 functional metagenomics expression library inserts [16]. Data for individually sequenced inserts (SRA accessions SRX375037 – SRX375128) where subjected to contaminant removal (against *E. coli* DH1, cosmid vector pJC8 and pRK7813 and hg38 reference assembly of the human genome sequence) and assembly with pipeline F1, employing the SPAdes assembler which is optimized for the assembly of small single genomes. In accord with the earlier work of Lam et al. [16], low sequencing coverage prevented useful assembly of 15 clones, while no end-tags were available for 4 other inserts. Lam et al. previously generated 2 end-tags for 61 of the remaining clones and a single end-tag for the remaining 12. We note that 3 successfully sequenced inserts represent overlapping loci derived from a single genome. As such these are expected to assemble as a single insert from pooled data and are accordingly treated herein as a single, merged, contig. Individual assembly of these 71 non-redundant clones for which end-tag Sanger sequences are available, yielded a single contig with the correct end-tags situated at both termini for 40 out of a possible 59) inserts, while 10 out 12 for which a single end-tag was available, allowed assembly of a contig with one expected terminal sequence. An additional 6 clones were identified in the form of 2 incompletely assembled insert fragments containing distinct terminal sequences matching the appropriate tags, while 11 assemblies allowed the identification of just a single, terminal end tag. Notwithstanding removal of possible contaminant sequences total size of individual assemblies of Lam et al. barcoded data, largely exceeded the nominal size of e-DNA insert (average nominal size = 33 Kb, average assembly size = 97 Kb, see Additional file 2: Figure S1 for a comparison of insert size distributions). In all cases assembled contigs did not show significant levels of similarity (average identity = 37%) with any of the possible known contaminant sequences, including vectors used for library construction, the host genome (*E. coli* DH1) and the human genome, thus suggesting widespread contamination of the libraries. For such reasons only the 67 complete and partial assemblies confidently assigned to end-tags (77 contigs, N50 = 36,113, total length = 229,845 nt) were subsequently used as a reference set to evaluate assemblies from pooled sequencing data (for a schematic description of these assemblies see Fig. 2).

Sequence data (SRA accession SRX367531), obtained by Lam et al. by pooling the 92 inserts prior to library construction and sequencing, were then assembled and annotated using the five pre-configured workflows provided with A-GAME as well as by two published metagenomics assembly and annotation pipelines MOCAT2 [20] and Parallel-META2 [21] (see Additional file 1).

The quality and completeness of each pooled assembly (as well as those generated by Lam et al. from the same data) was evaluated by comparison to the single insert reference assemblies using a series of metrics including:

1. The number of complete inserts with both Sanger end reads incorporated,
2. The number of inserts where both end-tags were detected at the termini of separate contigs,
3. The number of inserts for which only one end-tag was available and was found at the end of a contig,
4. The number of inserts for which only one of two end-tags was identified at the terminus of a contig,
5. Total size of end-tag containing contigs,
6. N50 of end-tag containing contigs,
7. Percentage of reference assembly represented in end-tag containing contigs,
8. Percentage of proteins annotated on the reference assemblies that provided reciprocal best BLAST matches to proteins annotated in each pooled assembly,
9. Computational resources employed (run time and peak RAM usage).

Summary statistics (Table 2) indicate that A-GAME workflows Fosmid1 (F1), Fosmid5 (F5) and Fosmid3 (F3) -which incorporate SPAdes, metaSPAdes and MEGAHIT respectively as assemblers- out-performed all other evaluated approaches with the current data. Unsurprisingly the F1 (based on SPAdes) and F5 (based on metaSPAdes) workflows, which are based on the same preprocessing strategy and similar short reads assembler programs (metaSPAdes is a version of the SPAdes program optimized for metagenomics assembly) attained nearly identical results, suggesting, that due to the generally low number of discrete and relatively small sequences that are usually pooled in one experiment, assembly and annotation eDNA inserts pools does not seem to benefit from sophisticated algorithmic approaches that are usually applied for *de-novo* assembly of high complexity metagenomics samples. Therefore, from here onward, we will refer to F1 and F5 as a single assembly pipeline: F1/5. F1/5 recovered a larger number of complete insert sequences with two terminal end-tags than F3, despite the observation that F3 assembled a larger proportion of the reference assembly as contigs with at least one terminal end-tag. This discrepancy is likely related to the fact that MEGAHIT, optimized for metagenomics assembly, excludes low frequency k-mers from the assembly graph (in order to constrain graph complexity), but uses additional heuristics to resolve assembly graph bubbles. MEGAHIT thus maximizes contiguity of assemblies in the presence of repetitive regions, but is highly sensitive to regions of low sequence coverage. MOCAT2 [18] yielded assemblies that were comparable to those initially generated by Lam et al. (both employ SOAPdenovo [22]), while workflow F2 outperformed Parallel-META2 despite both employing Velvet as assembler. Parallel-META2 provides a version of Velvet where only short k-mers may be used in De-Bruijn graph construction, producing sub-optimal assembly in the presence of short repeats when high sequence coverage is available, as in typical functional metagenomics data.

**Table 2:** Comparison of workflows for the assembly and annotation of eDNA insert data using Lam et al. [16] pooled inserts

	Insert assignment based on end-tags				Completeness			Computational requirements	
	Complete^a^	2 of 2 ends^b^	1 of 1 ends^c^	Partial^d^	% assembled^e^	% of reference proteins^f^	Assembly N50	CPU time (h)	RAM peak (Gb)
original assembly	40	6	10	11	100.00	100.00	36,113	NA	NA
SPAdes (F1)	34	13	9	11	88.16	86.35	34,329	2.03	5.3
Velvet (F2)	18	27	10	12	66.58	64.79	32,942	1.67	6.61
MEGAHIT (F3)	30	19	8	10	95.14	92.13	34,446	1.21	3.22
MetaVelvet (F4)	19	26	9	13	74.64	73.38	33,150	1.75	7.01
meta-SPAdes (F5)	34	13	9	11	88.16	86.35	34,329	2.03	5.3
MOCAT2	19	27	8	13	67.62	65.92	25,246	1.91	4.51
Parallel META2	12	34	6	15	40.48	37.87	26,408	2.36	3.11
Original from LAM et al	19	28	7	13	72.47	69.97	33,347	NA	NA

^a^Insert assembled into a single contig matching both end tags

^b^Inserts assembled into multiple contigs, both end tags are assigned

^c^Inserts for which only a single end tag is available and gets assigned

^d^Inserts for which both ends are available but only one is assigned

^e^Percentage of reference assembly represented in the pooled assembly

^f^Percentage of proteins from the reference assembly recovered in the pooled assembly

While we did not observe marked differences in computational requirements between all the pipelines tested in the course of the current study, we notice that the Fosmid3 workflow, which is based on the MEGAHIT metagenomics assembler, resulted to be the most efficient in terms of computational demands, achieving both the most rapid execution times and least usage of RAM memory. This is probably due to heuristics applied by MEGAHIT to discard low coverage data resulting in a more compact and easy to navigate assembly graph. F1/5 required the longest execution time among the workflows implemented in A-GAME, the differences however are in large part due the error correction of reads performed by SPAdes requiring approximately 35 min. Mocat-2 requirements were very similar to F1/5, while parallel-META2 was slightly more demanding both in terms of execution times and memory usage.

Scaffolding contigs using Paired-End sequence data, SSPACE [23] was unable to improve any assemblies from the current data, principally due to the small insert sizes of the current libraries (mean insert size =285 bp).

The highly significant correlation between percentage of reference assembly recovered in end-tagged contigs and the percentage of “reference proteins” annotated (R^2^ = 0.98, *p*-value = 1.096e-07) strongly suggests that contiguity of assembly - rather than fundamental differences in annotation quality by different methods – determines reference protein discovery in this experiment.

Equivalent comparative analyses of the workflows performed on simulated datasets (see Additional file 1 and Additional file 3: Table S3) are highly consistent with our main finding suggesting that, notwithstanding the satisfactory results achieved by all the pipelines, the F1/F5 workflow (or equivalent workflows based incorporating SPAdes or metaSPAdes) should represent suitable starting points for the assembly of eDNA insert data within A-GAME.

### The FosBin tool

Several tools have been developed to group, or bin, WGS metagenomics contigs into candidate “single genome” clusters [24–27]. Such methods typically rely on the relative frequencies of short k-mers in assembled contigs, the depth of sequencing coverage and the assumption that a “complete” genome sequence should contain a set of “core” genes. While in WGS metagenomics the “core genes” approach can be used both to establish the optimal number of clusters into which the meta assembly should be partitioned and to assist in the allocation of contigs to bins, it is fundamentally inappropriate for eDNA insert sequences where a complete set of core genes is unlikely to be present in each sub-genomic insert. We have implemented an alternative method (named FosBin, see methods), which uses K-means clustering (as implemented in the R library Cluster) of tetranucleotide frequencies and k-mer coverage values (extracted from the output of each of the included assemblers) for each contig. FosBin is available in A-GAME and is included in the A-Game package available in the main Galaxy toolshed.

We simulated datasets representing incomplete assemblies of 8, 12 or 18 pooled inserts by randomly selecting inserts with 2 associated end-tags from barcoded assemblies of the Lam et al. data described above. The inserts in each pool were randomly fragmented into 2, 3 or 5 contigs, whose k-mer coverage values were calculated by re-mapping reads derived from Lam et al. pooled sequencing experiment to the fragmented contigs. Each combination of pool size and fragmentation was independently simulated 100 times and resulting pools were subjected to clustering using FosBin. We evaluated sensitivity (the proportion of contigs assigned to a cluster containing at least one other contig from the same insert), and specificity (the proportion of clusters containing only sequences from the same insert). Results (Table 3) suggest that while the majority (c. 90%) of clusters recovered by FosBin consist of contigs from single inserts, a proportion of fragments are consistently recovered in clusters lacking other contigs from the same insert. Evaluation of the characteristics of” correctly” and “incorrectly” clustered contigs indicated that the latter were significantly shorter than the former (typically less than 2Kb in length) and exhibited lower coverage than correctly clustered contigs (Additional file 4: Figure S2).

**Table 3:** Sensitivity and specificity of the FosBin tool with real coverage and simulated coverage levels

		Real coverage		Simulated coverage	
N° of fosmids^a^	N° of fragments^b^	Sensitivity	Specificity	Sensitivity	Specificity
8	2	0.748	0.874	0.808	0.904
8	3	0.766	0.922	0.822	0.941
8	5	0.713	0.943	0.779	0.956
12	2	0.717	0.858	0.782	0.891
12	3	0.726	0.909	0.790	0.930
12	5	0.701	0.940	0.769	0.954
18	2	0.656	0.828	0.730	0.865
18	3	0.635	0.878	0.711	0.904
18	5	0.589	0.918	0.670	0.934

^a^Number of inserts included in the simulated pool

^b^Number of distinct fragments generated from each insert

A further simulation, wherein coverage values for each insert were randomly multiplied by 2, 4, 8 or 16 – in order to simulate greater variety of sequencing depth between inserts – resulted in improved sensitivity and specificity (Table 3, and see discussion).

### Tools for functional annotation

Annotation and characterization of predicted proteins for the identification of candidate genes with enzymatic activities of interest from metagenomis eDNA screen requires careful annotation of predicted ORFs and identification of their functional domains. This process is often performed manually and requires a substantial amount of work, possibly including similarity searches against curated collections of prokaryotic proteins. A-GAME offers dedicated tools and resources that can assist in the functional annotation of predicted ORFs saving the need for laborious manual work.

A comprehensive yest simple report of PFAM domain annotations for the predicted proteins can be generated using the dedicated custom tool in A-GAME. The report consists of a fasta-like html page, where protein sequences are reported with corresponding PFAM domains and a concise, textual, description retrieved directly from the PFAM database by parsing the “pfamA.txt” file (*ftp://ftp.ebi.ac.uk/pub/databases/Pfam/releases/Pfam31.0/Pfam-A.full.gz*). Hyperlinks to PFAM are included in the report to facilitate the retrieval of additional information regarding domain activity and structure. For datasets where contig clustering (FosBin) or Sanger end-tag data are available, annotation of each inferred cluster is reported in a dedicated html page. Users can navigate through individual reports using hyperlinks provided at the top of the main page. Moreover, proteins containing functional domains of interest can be retrieved by performing keyword searches of PFAM domain descriptions. Multiple keywords can be specified and combined using the logical connectors, AND, OR and NOT.

**Fig. 3:**
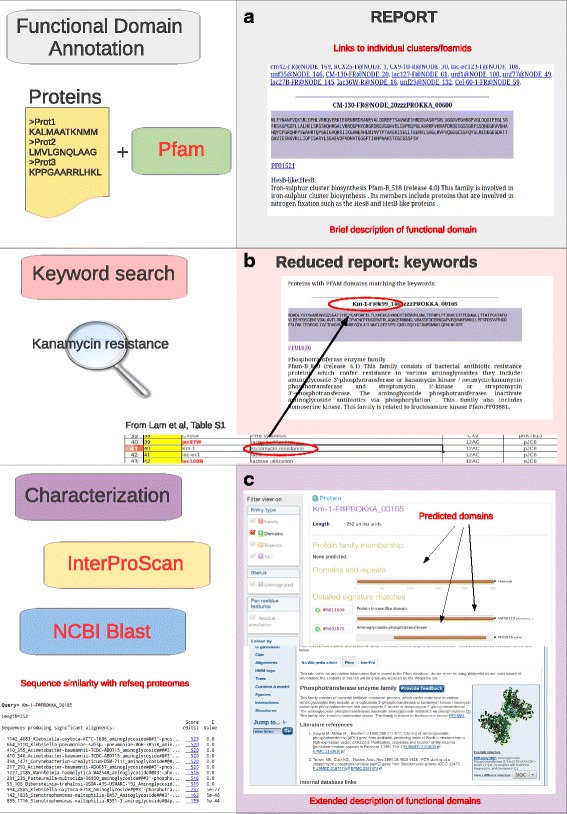
Identification of candidate genes/protein a. A-GAME offers a collection of tools and utilities for the functional annotation of proteins that can be used in order to facilitate the identification of genes with enzymatic activity of interest. **a** Concise report of PFAM protein domain annotation. The report is presented as an html page in a fasta-like format. Protein sequences are associated to their PFAM domains and a brief description. Hyperlinks to PFAM are included in the report in order to facilitate the retrieval of more complete information. For datasets where contig clustering (FosBin) or Sanger end-tag data are available, annotation of each inferred cluster is reported in a dedicated html page. Users can navigate through individual reports using hyperlinks provided at the top of the main page. **b**. Keyword search of PFAM domain annotation. Candidate genes with enzymatic activities of interest can be retrieved by performing keyword search of PFAM domain annotation by using the dedicated utility in A-GAME. Protein matching user provided keywords are reported in a dedicated report. **c**. Characterization of candidate proteins. The Interpro suite can be used for more specific annotation of functional domains in user selected subsets of genes to generate pages containing graphical depictions with descriptions and web-links to corresponding databases. A-GAME allows users to perform similarity searches against a local database containing more than 2500 Refseq bacterial proteomes to identify homologs of reconstructed ORFs

Selected proteins can be subjected to more thorough functional annotation of protein domains using the InterPro suite [28]. Sequences similarity searches of predicted ORFs against the non-redundant protein database [29] or a local database of over 2500 refseq bacterial proteomes can be performed, by the means of BLASTP, in order to refine the annotation and assess the similarity with “known” proteins. An example of a workflow for the functional annotation and characterization of proteins of interest is provided in Fig. 3, where we demonstrate the retrieval of clones selected for kanamycin resistance from the Lam et al. dataset.

## Discussion

The advent of high next generation DNA sequencing (NGS) technologies and the associated reduction in sequencing costs has contributed to the development of functional metagenomics approaches for the identification of genes and biosynthetic pathways with potential for biotechnological exploitation. However, analyses of NGS sequencing data can be complex and time-consuming and typically requires specialist intervention by bioinformaticians. At the present time, we are unaware of tools or pipelines specifically developed for the analysis of functional Metagenomics sequence data, while available shotgun metagenomics software tools typically lack a graphic user interface and exhibit limited potential for customization.

Here we have introduced A-GAME, a Galaxy web server that provides selected tools and pre-configured workflows for the assembly and annotation of eDNA sequence data. We demonstrate the application of A-GAME to a real case study, illustrating its improvement over classical metagenomics pipelines for the assembly and annotation of functional metagenomics data. We show that FosBin, a simple tool to group contigs from incompletely assembled inserts, performs well for the assignment of longer contigs (>c. 2 kb) indicating that, in conjunction with Sanger end-tags, it can be used to assist in predicting the clonal origin of incompletely assembled inserts.

Depending on the scale and objectives of functional metagenomics projects, several aspects of the experimental execution as well as design of the sequencing strategy might be exploited to further improve assembly results. Even where measures to screen contaminant sequences are employed, the assembly process benefits from high purity of DNA sequencing templates. Indeed, even in the single insert assemblies generated here from the barcoded sequencing of Lam et al. we observed the assembly of contigs originating from contaminants of both bacterial and non-bacterial origin. As well as complicating assembly, such contaminants detract from the accuracy of estimates of insert concentration for library production and mixing. Analogously, accurate information regarding the number of pooled inserts is important for FosBin which requires a-priori specification of the number of clusters to generate. While this last assertion might seem obvious, we note that several of the barcoded libraries generated by Lam et al. likely contain multiple eDNA inserts (Additional file 2: Figure S1). Taken together, these considerations underline the fact that the most sophisticated assembly and annotation methods are constrained by the quality of the data provided. We further note that artificially increasing the discrepancy between sequencing depths of distinct inserts has a positive effect on the capacity of FosBin to accurately cluster incompletely assembled inserts. In this light we suggest that, given high purity template DNA, Sanger sequences from insert ends might be used to estimate GC content of individual inserts and manipulate concentrations of inserts during library construction, such that inserts with similar composition are less likely to share similar coverage.

## Conclusions

In summary, we have shown both that choice of preprocessing and assembly steps can greatly influence the quality of assembly and annotation of pooled insert sequence data and that preformatted workflows in A-GAME outperform pipelines designed for shotgun metagenomics in this context. A-GAME also provides dedicated tools for clustering of non-contiguously assembled inserts and exploration of functional annotations; facilitating identification, prioritization and isolation of candidate genes for biotechnological exploitation. Accordingly, we believe A-GAME will constitute a valuable resource for the functional metagenomics community.

## Availability and requirements

Project name: A-GAME.

Project home page: e.g. http://beaconlab.it/agame

Operating system(s): Platform independent.

Programming language: Python.

Other requirements: none.

License: MIT License, (https://github.com/mpg-age-bioinformatics/galaxy-admin/blob/master/LICENSE).

Any restrictions to use by non-academics: no restriction.

## Additional files

Additional file 1: Supplementary methods and results. (DOC 52 kb)

Additional file 2: Supplementary Figure 1. (PDF 4 kb)

Additional file 3: Supplementary Tables (1 to 3). (XLS 32 kb)

Additional file 4: Supplemetary Figure 2. (PDF 113 kb)

## Abbreviations

BAC: Bacterial artificial chromosome
eDNA: environmental DNA
GS: genome specific coverage
HC: high uniform coverage
NGS: Next Generation Sequencing
ORF: open reading frame
RN: random coverage.
WGS: whole genome shotgun
