# Supplementary material for: A-GAME: improving the assembly of pooled functional metagenomics sequence data
Source: BMC Genomics. 2018 Jan 12;19:44. doi: 10.1186/s12864-017-4369-z (PMC5767027; doi:10.1186/s12864-017-4369-z)
Supplement: Supplementary file 1 — Supplementary methods and results. (DOC 52 kb) [file 12864_2017_4369_MOESM1_ESM.doc]

**Supplementary Methods**

A-GAME is based on Galaxy release 16.07. The Galaxy framework was downloaded from the official Galaxy repository on github (<https://github.com/galaxyproject>). The complete list of tools incorporated into a A-GAME is reported in Table 1 (main text). Where possible, tools were installed directly from the official Galaxy tool-shed repository. Galaxy wrappers were prepared ad-hoc for PFAM_scan, primer3, and MaxBin.

A-GAME also features various custom utilities, including a quality trimmer for pre-processing of sequencing reads, and a set of programs designed to assist in analysis of eDNA data. These post-processing tools include Fosbin, a program to cluster incomplete contigs obtained from pooled eDNA libraries to a probable common fosmid origin as well as a blast-based tool for the assignment of contigs to eDNA inserts (where Sanger sequencing of fosmid- ends has been performed), a tool for keyword searching of PFAM annotations, and a tool to generate a data rich output from PFAM annotations. All the custom utilities were implemented using the Perl programming language, apart from the quality trimmer which is implemented in C++ and the tool for the clustering of the contigs which is based on the R implementation of the K-means algorithm.

**2.1 Parsing of PFAM annotation and generation of an html report**

The custom tool for the processing of the PFAM annotation is a simple text parser based on regular expressions that can associate the synthetic description related to each domain in the database, as reported in the pfam domain description file or in the clans description file - for domains belonging in clans - with the identifier of the domains. Description files are regularly downloaded from pfam.xfam.org. The tool automatically generates an html report containing a table with the synthetic description as well as hyperlinks to the corresponding entry in the PFAM database. The report is presented in a pseudo fasta format, where the descriptions are associated with the predicted protein sequences. Only proteins containing a PFAM domain are included in the report.

**2.2 Keyword searches within PFAM domains description**

Keyword searches are performed through a simple Perl script implementing exact searches of user specified keywords within the description of each PFAM domain as reported in the clans.txt or pfam.txt domain description files. Searches can be performed using logical connectors, AND, OR and NOT. When multiple keywords are entered, the default is to use the AND connector.

**2.3 Blast-based assignment of contigs to eDNA inserts**

The blastn program from the blast+ suite is used to perform sequence similarity searches of the fosmid termini sequences against a reference database containing the fosmid assembly. A custom Perl script is used to parse the results of the alignment and assign the contigs to the presumed inserts. Only alignments showing levels of identity higher than a user defined cut-off (default 95%) and covering more than a user defined proportion of the Sanger sequence (default more than 60%) are considered. The final output consists of a multi-fasta file where the information containing the presumed insert assignment is added to the header of each sequence.

**2.4 Fosbin - Kmeans clustering of contigs**

Clustering of contigs is performed by a custom script based on the R implementation of the K-means algorithm, using 1500 starting positions for the centroids. The clustering is performed on metrics based on coverage, GC composition and tetra-nucleotide composition of each contig, which are computed directly from the fasta file. The user must input the desired number of clusters, contigs are partitioned accordingly.

**2.5 Custom algorithm for the quality trimming of sequencing reads**

Our custom script for quality trimming implements strict quality filters based on the provided base call quality scores. Reads are iteratively trimmed from the 3′ end until all of the following conditions were satisfied:

1. the median quality score (Qscore) of upstream bases is ≥15
2. less than 10 bases with Qscore ≤10 and less than 15 bases with Qscore ≤ 20 are present in the upstream sequence
3. the cumulative error probability in the upstream region is below a user defined cutoff
4. the length of the trimmed read exceeds a user defined cutoff

The program is designed to work with paired end sequencing files only, the output consists in 3 distinct files containing the pairs where both mate passed the filters (output to 2 separate files) and a third file containing all the singleton reads, for which the corresponding mate did not pass the quality filters.

**2.6 Installation and configuration of Mocat-2 and parallel-meta**

The MOCAT2 assembly workflow uses fastX (https://github.com/agordon/fastx_toolkit) for quality trimming of the reads, Usearch [1] and bwa [2] for contaminant filtering against a reference collection of possible contaminant sequences, Soapdenovo2 [3] for genome assembly and Prodigal [4] and Metagenemark [5] for gene prediction. Functional annotation is performed by an heuristic method that incorporates 18 carefully selected functional databases. Predicted genes can be clustered by similarity into a reference gene catalog which is mapped to the eggNOG [6] database to which sequence annotations from other databases have been pre-computed. Parallel-META2 uses Velvet [7] for genome assembly and Fragenescan [8] for gene prediction. Functional annotation of the predicted genes is performed by sequence similarity searches against the SEED databases [9]. No tools for contaminant filtering and quality trimming of the reads are incorporated in this pipeline, although for our evaluation, data were subjected to vector and contaminant filtering (*E. coli* DH1, cosmid vector pJC8 and pRK7813 and hg38 reference assembly of the human genome sequence) prior to submission to the pipeline.

Mocat-2 and Parallel-META2 were downloaded from their respective repositories and used with their default parameters. In our hands installation of the programs required 2 and 3 hours respectively. Mocat-2 was downloaded from http://mocat.embl.de/download.html, the program consists of a series of scripts, that perform different tasks (contaminant filtering, assembly annotation, similarity searches) all tied together by a main script that serves as the (command line) interface. The software is implemented in Perl and Python, and it is solely designed to be used on 64 bits Unix systems. Mocat-2 comes with a convenient configuration script that facilitates the installation and enables the download of the relevant databases and example datasets. While most of the programs used by Mocat-2 are already included in the main distribution, to get full functionalities users are required to obtain and manually install a copy of Usearch [1] and MetaGeneMark [5].

Parallel-META2 was downloaded from <https://github.com/Comp-Bio-Group/Parallel-META>. Like Mocat-2, this program is specifically designed for the Unix environment and has a command line interface. Parallel-META2 doesn't have a configuration script, and a few software packages (namely the blast-suite [10], velvet [7] and FraGenescan [8] need to be installed manually in order to generate a fully functional version of the program.

**Supplementary results**

**Evaluation of assembly pipelines on simulated data**

In order to assess the performances of the various assembly pipelines and tools tested in the course if the current study, we performed an extensive simulation of the sequencing of a large pool of fosmids derived from 9 genomes, selected for having a wide composition range, from 32% to 67% C+G (Table S1). The fosmids were simulated by randomly selecting 35 and 40 Kbp regions of the Refseq assembly of these genomes, and by recovering the corresponding reads from their respective WGS shotgun project entries in the SRA archive. The simulated pools contained 61 fosmids respectively and multiple fosmids (5 to 8) were simulated from each genome. Full details are reported in Table S2. To evaluate the effect of coverage unevenness on the assembly and annotation we simulated reads for each pool under three different coverage scenarios:

- High uniform coverage (HC): all the fosmids have a theoretical 150x coverage
- Genome specific coverage (GS): all the fosmids originating from the same genome have the same coverage, coverage ranges are in between 45 to 135x
- Random coverage (RN): coverage ranges from 2 to 150x

The 61 inserts were subjected to assembly, gene prediction and functional annotation using the Fosmids pipelines available through A-GAME, Mocat-2 or parallel-META2. All the workflows were run on the same machine (a unix server with 12 intel Xeon CPU cores and 64 Gb of RAM) in single processor mode. Results were evaluated in terms of both contiguity and accuracy of the assemblies by comparing them to the corresponding genomic intervals from which the artificial fosmids were derived. Assembly accuracy was evaluated in the light of the average number of single nucleotide variants and indels (that are likely to represent assembly errors) incorporated into the final assembly obtained by each pipeline. The annotation was evaluated by considering the proportion of Refseq genes that were recovered. Results are summarized in Table S3.

Unsurprisingly the F1 (based on SPAdes) and F5 (based on metaSPAdes) pipelines attained nearly identical results also in this simulation, consistent with what was already observed for real data. Therefore, from here onward, Fosmid 1 and Fosmid 5 will be referred to as a single assembly pipeline Fosmid 1/5.

In our simulations, the Fosmid 1/5 pipeline achieved the best results both in term of accuracy (lower indel and SNP rates) and contiguity (overall highest N50 and N90) of the assembly. This is particularly evident in the case of the RN datasets, where the assemblies obtained by this pipeline are more complete and contiguous with respect to those attained the other pipelines. As a direct consequence of the quality of the assembly, we observe that annotations obtained by the Fosmid1/5 workflow are in general more complete and contain a higher proportion of Refseq genes with respect to the alternative methods. This is mostly due to the difficulty of predicting genes in highly fragmented assemblies but also to the fact that the Fosmid1/5 assemblies contain less sequencing/assembly errors, and indels in particular, and is highly consistent with similar observations from the analysis of actual eDNA inserts sequencing (see Table 2 in the main paper). Taken together these findings would suggest that the assembly algorithm implemented in SPAdes, which employs multiple de Brujijn graphs of different orders, seems well suited to the assembly of pooled eDNA clones as it should be capable of accommodating different sequencing depths-resulting from both compositional factors and differing proportions in which clones might be mixed. Accordingly, we consider that, the satisfactory results achieved by all the pipelines notwithstanding, the F1/F5 workflow (or equivalent workflows based incorporating SPAdes or metaSPAdes) should represent suitable starting points for the assembly of eDNA data within A-GAME.

**General evaluation of the assemblies and utilities available through A-GAME**

Although we could not observe any mis-assembly within any of the pipelines used in the course current study, results of the assembly of eDNA inserts, (and indeed of any short-read assembly) should be evaluated with care in order to understand whether they reflect an accurate representation of the actual genomic fragments that were selected and subjected to sequencing or they are likely to contain assembly errors or mis-assemblies. A-GAME offers several tools and utilities that can be used in order to facilitate this process.

If Sanger end tags, derived from the sequencing of the inserts termini are available (as recommended to users of A-GAME), users can take advantage of the “Sanger Ends Attacher” tool (see point 2.3 above) in order to verify whether presumably complete assemblies of the inserts are matched consistently with their ends. Even if this strategy is not 100% robust in the presence of a fragmented assembly, it can be used as a first criterion to identify possible chimeric sequences.

Furthermore, in the presence of fragmented assemblies, results from the Sanger Ends Attacher can be subjected to downstream analyses using FosBin and information related to the insert termini can be used to (partially) assess the correctness of proposed clusters: a cluster of fragments including 2 corresponding end-tags and with an expected total length is a good (but not perfect) indication of “correctness”.

It is expected that preliminary analyses of clones subsequently pooled for sequencing and analysis by A-GAME would include estimates of insert size. In this scenario, since no expectation of insert size is given to the assembler(s), an insert assembly of the expected size containing the expected end-tags is evidently, in itself, a good indication of correctness of assembly.

Users could also eventually search for synteny of predicted proteins with published sequenced genomes using BLAST or equivalent tools. A-GAME allows users to perform similarity searches against a local database containing more than 1700 Refseq bacterial proteomes to identify homologs of reconstructed ORFs. Output and alignments can be formatted in various formats.

Finally, chimerism in the assembly can be identified by using strategies based on compositional analysis of the sequences. Since bacterial genome composition is usually very homogeneous across short genomic regions, such as those that are typically concerned in eDNA inserts assembly, sudden compositional shifts might reflect inconsistencies or erroneous assemblies. While several tools can be used in principle to perform this type of analysis, some of which are also publicly available through the official Galaxy tool registry, A-GAME offers its own dedicated utility (GC-plotter) to facilitate the visual inspection of GC composition of DNA sequences along windows of user specified length.

**Supplementary References**

*[1] Edgar RC. Search and clustering orders of magnitude faster than BLAST. Bioinformatics. 26(19):2460-1. doi: 10.1093/bioinformatics/btq461. (2010)*

*[2] Li H, Durbin R. Fast and accurate short read alignment with Burrows-Wheeler transform. Bioinformatics. 25(14):1754-60. doi:10.1093/bioinformatics/btp324. (2009)*

*[3] Li R, Zhu H, Ruan J, Qian W, Fang X, Shi Z, Li Y, Li S, Shan G, Kristiansen K,Li S, Yang H, Wang J, Wang J. De novo assembly of human genomes with massively parallel short read sequencing. Genome Res. 20(2):265-72. Doi: 10.1101/gr.097261.109. (2010)*

*[4] Hyatt D, Chen G-L, LoCascio PF, Land ML, Larimer FW, Hauser LJ. Prodigal: prokaryotic gene recognition and translation initiation site identification. BMC Bioinformatics. 11:119. doi:10.1186/1471-2105-11-119. (2010)*

*[5] Zhu W, Lomsadze A, Borodovsky M. Ab initio gene identification in metagenomic sequences. Nucleic Acids Res.38(12): e132. doi: 10.1093/nar/gkq275. (2010)*

*[6] Huerta-Cepas J, Szklarczyk D, Forslund K, Cook H, Heller D, Walter MC, Rattei T, Mende DR, Sunagawa S, Kuhn M, Jensen LJ, von Mering C, Bork P. eggNOG 4.5: a hierarchical orthology framework with improved functional annotations for eukaryotic, prokaryotic and viral sequences. Nucleic Acids Res. 44(D1):D286-93. doi: 10.1093/nar/gkv1248. (2016)*

*[7] Zerbino DR, Birney E. Velvet: Algorithms for de novo short read assembly using de Bruijn graphs. Genome Research. 18(5):821-829. doi:10.1101/gr.074492.107. (2008)*

*[8] Rho M, Tang H, Ye Y. FragGeneScan: predicting genes in short and error-prone reads. Nucleic Acids Res. 38(20):e191. doi: 10.1093/nar/gkq747. (2010)*

*[9] Overbeek R, Begley T, Butler RM, Choudhuri JV, Chuang HY, Cohoon M, de Crécy-Lagard V, Diaz N, Disz T, Edwards R, Fonstein M, Frank ED, Gerdes S, Glass EM, Goesmann A, Hanson A, Iwata-Reuyl D, Jensen R, Jamshidi N, Krause L, Kubal M, Larsen N, Linke B, McHardy AC, Meyer F, Neuweger H, Olsen G, Olson R, Osterman A, Portnoy V, Pusch GD, Rodionov DA, Rückert C, Steiner J, Stevens R, Thiele I, Vassieva O, Ye Y, Zagnitko O, Vonstein V. The subsystems approach to genome annotation and its use in the project to annotate 1000 genomes. Nucleic Acids Res. 33(17):5691-702. (2005)*

*[10] Camacho C, Coulouris G, Avagyan V, Ma N, Papadopoulos J, Bealer K, Madden TL. BLAST+: architecture and applications. BMC Bioinformatics. 15;10:421. doi: 10.1186/1471-2105-10-421. (2009)*
