# Supplementary figures and images for: A-GAME: improving the assembly of pooled functional metagenomics sequence data
Source: BMC Genomics. 2018 Jan 12;19:44. doi: 10.1186/s12864-017-4369-z (PMC5767027; doi:10.1186/s12864-017-4369-z)

**Nominal insert vs Total Assembly Size**

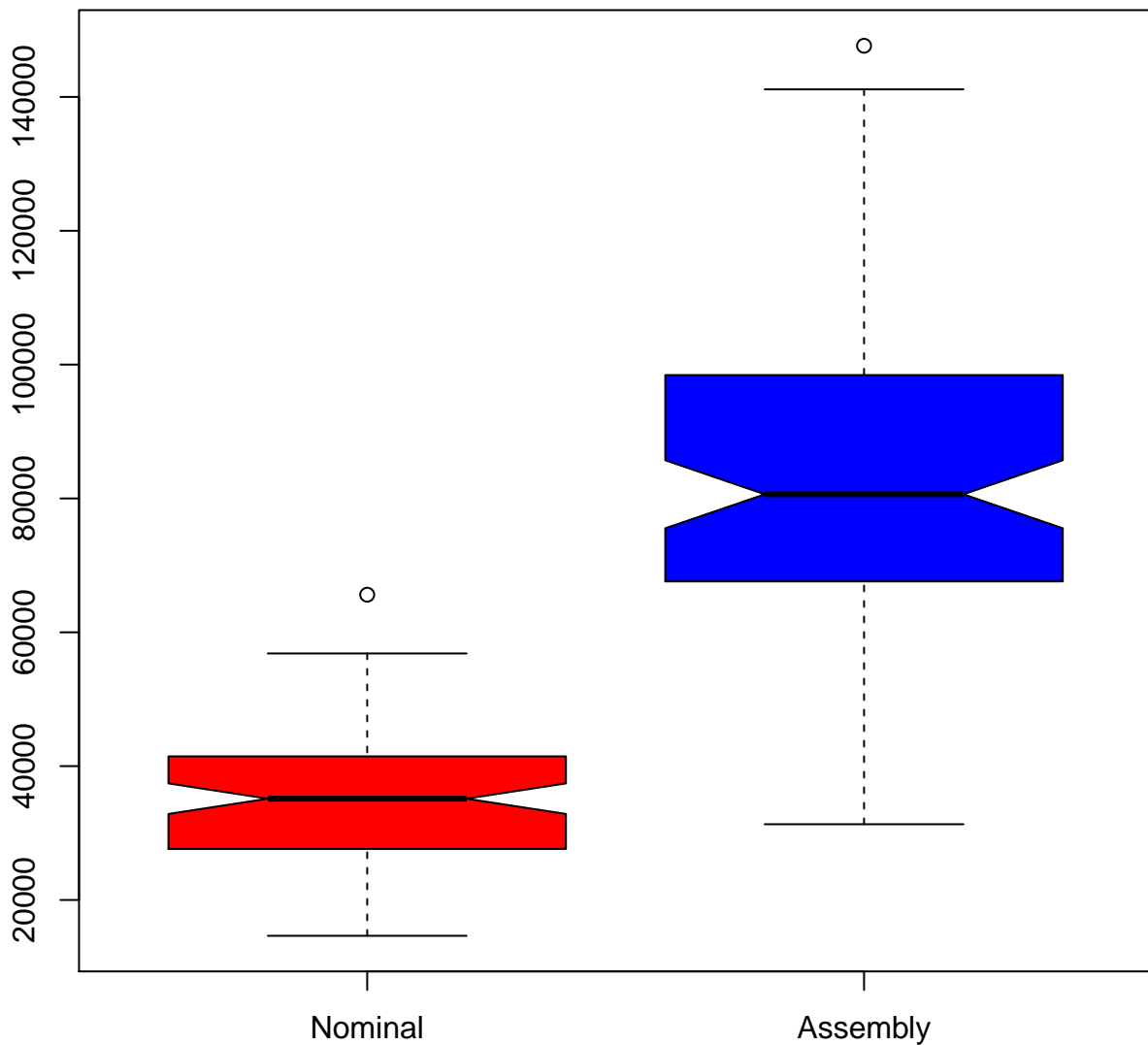

Supplement: Supplementary file 2 — Supplementary Figure 1. (PDF 4 kb) [file 12864_2017_4369_MOESM2_ESM.pdf]

A.

Average size

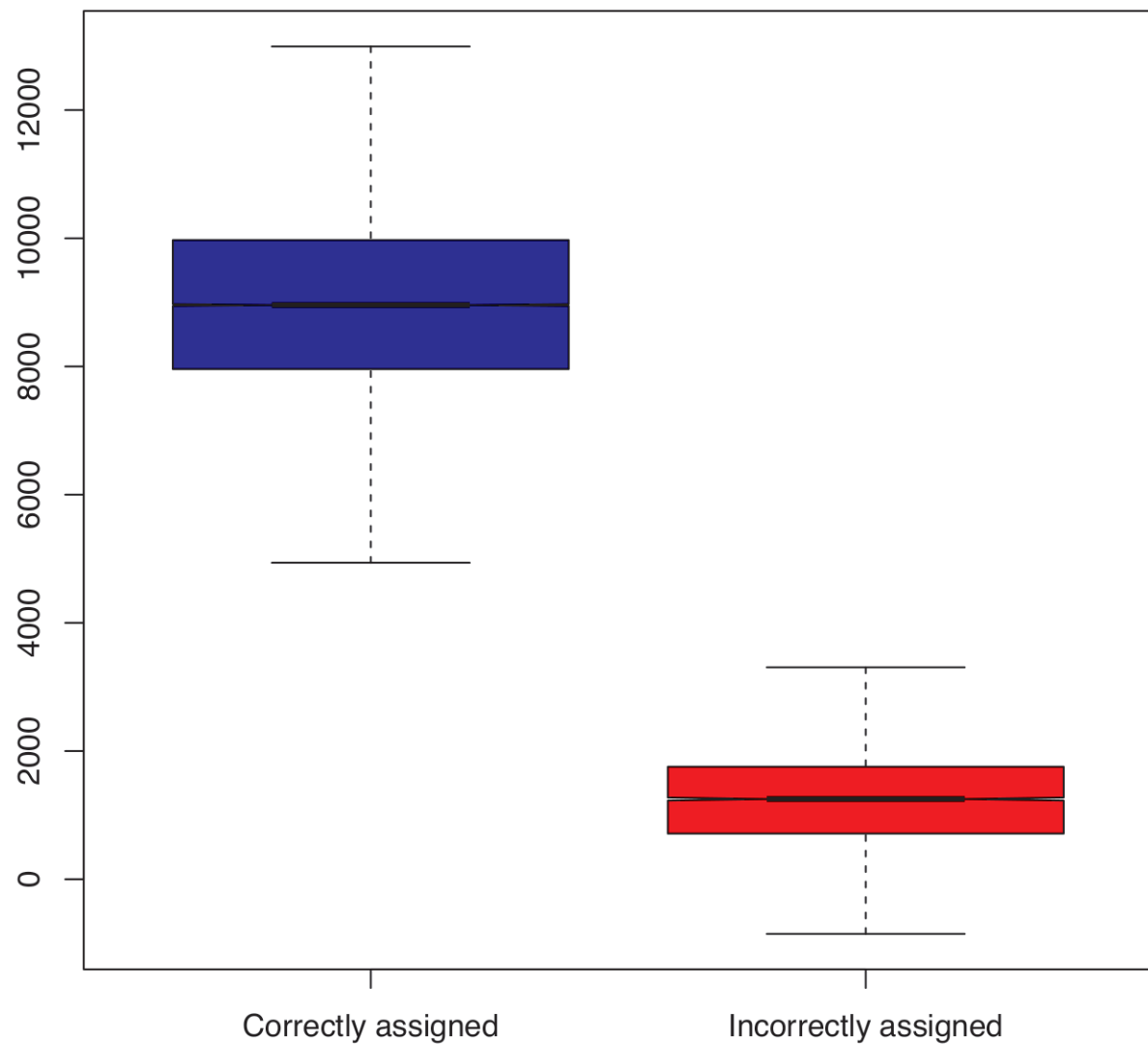

B.

Avg reads reads x 100 bp

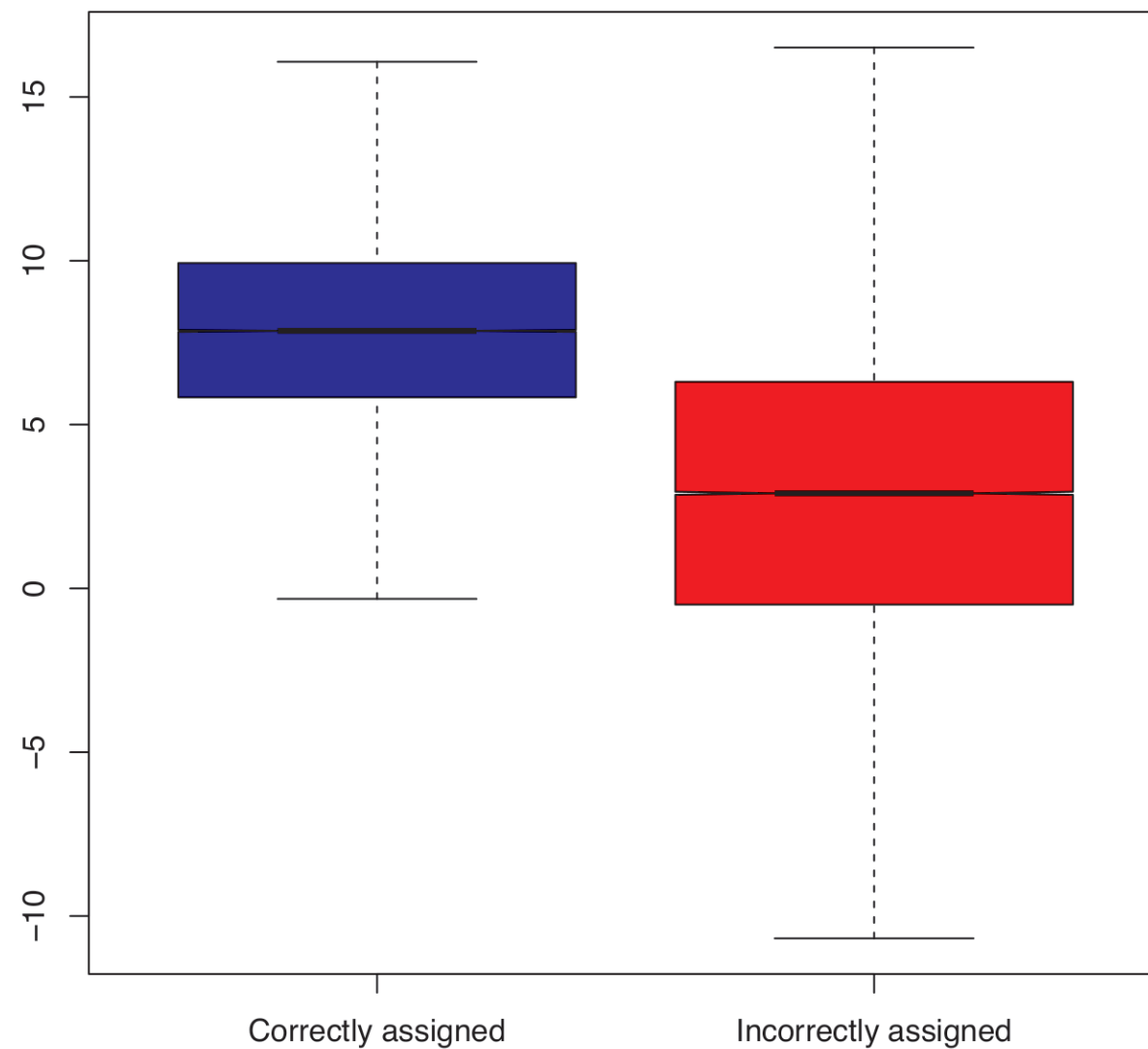

Supplement: Supplementary file 4 — Supplemetary Figure 2. (PDF 113 kb) [file 12864_2017_4369_MOESM4_ESM.pdf]
